# Recycling-Oriented Characterization of Post-Earthquake Building Waste by Different Sensing Techniques

**DOI:** 10.3390/jimaging7090182

**Published:** 2021-09-08

**Authors:** Oriana Trotta, Giuseppe Bonifazi, Giuseppe Capobianco, Silvia Serranti

**Affiliations:** Department of Chemical Engineering, Materials & Environment, Sapienza University of Rome, Via Eudossiana 18, 00184 Rome, Italy; oriana.trotta@uniroma1.it (O.T.); giuseppe.capobianco@uniroma1.it (G.C.); silvia.serranti@uniroma1.it (S.S.)

**Keywords:** hyperspectral imaging, construction and demolition waste, post-earthquake building waste, micro-X-ray fluorescence, degree of liberation, quality control, recycled masonry aggregate, cement mortar, tile

## Abstract

In this paper, a methodological approach based on hyperspectral imaging (HSI) working in the short-wave infrared range (1000–2500 nm) was developed and applied for the recycling-oriented characterization of post-earthquake building waste. In more detail, the presence of residual cement mortar on the surface of tile fragments that can be recycled as aggregates was estimated. The acquired hyperspectral images were analyzed by applying different chemometric methods: principal component analysis (PCA) for data exploration and partial least-squares-discriminant analysis (PLS-DA) to build classification models. Micro-X-ray fluorescence (micro-XRF) maps were also obtained on the same samples in order to validate the HSI classification results. Results showed that it is possible to identify cement mortar on the surface of the recycled tile, evaluating its degree of liberation. The recognition is automatic and non-destructive and can be applied for recycling-oriented purposes at recycling plants.

## 1. Introduction

Earthquakes often generate a large amount of debris and waste. In some cases, the amount of debris from a single event is five to fifteen times greater than the amount of waste normally generated in a year in the affected regions [1]. Waste streams generated during disasters include vegetative or green waste, sediments/soil and rocks, hazardous waste (i.e., oils, pesticides, etc.), construction and demolition waste (CDW) from damaged buildings and infrastructure (such as roads, houses, and other facilities), industrial and toxic chemicals (including fuel products), vehicles, plastics, metals, electronics, and white goods [2]. Nowadays, the main challenge is the disposal of this massive and diverse type of debris, as well as the reuse and recycling of this material due to the lack of resources for post-earthquake reconstruction. Therefore, special attention must be paid to finding appropriate solutions for debris recycling, in order to obtain secondary raw materials whose properties are comparable to those of primary raw materials [3]. In particular, the use of recycled aggregates (RA) for the production of new concrete has become more common in recent years [4]. However, the main use of RA from CDW is still for unbound road base or as structural layer material. These applications bring low added value to these materials [5]. In order to reuse RA for the production of new concrete, the mechanical and durability properties must be the same as those of natural aggregates [6]. In recent years, the mechanical and chemical properties of RA made from CDW have been extensively studied, showing the possibility of replacing natural aggregates [7,8]. Many studies highlight that RA is mostly composed of recycled concrete aggregates (RCA), recycled masonry aggregate (RMA), and mixed recycled aggregate (MRA) [6]. The most used RA for concrete production are RCA; however, recent studies show the potential of RMA for this purpose [9,10]. Within the class of RMA, tiles have special proprieties as they are durable, hard, and highly resistant to biological, chemical, and physical degradation processes [11,12,13,14,15,16]. However, as for RCA aggregates, the reuse of tile in concrete production requires an evaluation of the presence of residual aged cement mortar on the surface of RA. More specifically, the presence of cement mortar can affect density, water absorption, sulfate content, consistency, and strength of the aggregates and thus the performance of the newly produced concrete [17]. For these reasons, before recycling an aggregate, various mechanical methods can be used to separate the cement mortar, such as autogenous cleaning, ball/tube milling, impact crushing, eccentric shaft rotor, and screw abrading. Despite the application of these methods, experimental results show that aggregates are never completely cleaned and/or can be damaged by the applied methods [18]. Thus, no method is 100% satisfactory, since these treatments can be affected by an error. Therefore, quality control techniques that certify the properties of the products are required to select RA according to their target uses [19]. Hyperspectral imaging (HSI) can be a valuable tool for non-destructive, objective, and full in-line quality control of construction debris after an earthquake. Several studies have shown how it is possible to detect different types of debris from a post-disaster scenario. In particular, the study demonstrated the capability of HSI to recognize and classify different categories of material, i.e., wood [20,21], asbestos-containing materials [22], demolition debris [23,24,25], furniture [26], vegetative debris [27,28], plastic waste materials [29,30,31,32,33], mixed metals [34,35,36,37], etc.

More specifically, the HSI technique, combining imaging with spectroscopy, can be useful for the investigation of the above-mentioned materials [38,39]. HSI, coupled with chemometric methods, allows for the obtainment of information on the physical-chemical characteristics of the investigated materials and their nature, thanks to the 3D nature (i.e., spectral and spatial) of hyperspectral data [40].

Nowadays, the main difficulty in a post-earthquake flow stream is to evaluate the quality of recovered materials characterized by similar chemical composition, but presenting different mechanical proprieties, these latter strongly influencing their correct reuse: the presence of cement mortar in recovered inert products (i.e., aggregates, tiles, bricks, etc.) can dramatically influence the possibilities of their correct reuse as recycled aggregate. Concerning RA, previous studies have demonstrated the possibility of evaluating building waste by HSI, assessing the presence of contaminants in RA, and classifying end-of-life construction materials [41,42,43,44]. However, few studies have been carried out on the monitoring of tiles coming from post-disaster building waste and on their possible reuse as RMA, despite their high presence in earthquake-damaged structures. For this reason, in this work, the evaluation of the degree of liberation of tile fragments from cement mortar was performed by HSI coupled with a machine learning approach. More specifically, the acquired hyperspectral data were first examined using principal component analysis (PCA). Then, a classification model, based on partial least squares-discriminant analysis (PLS-DA) was applied to detect cement mortar covering the tile and the relative degree of liberation. The same samples underwent preliminary investigation by micro X-ray fluorescence (micro-XRF) to obtain the chemical element maps showing the distribution of cement mortar on the surface of tile fragments. The micro-XRF maps were then compared with the corresponding maps generated by hyperspectral modelling. The improvement of this strategy, able to recognize and topologically evaluate the detected contaminants, can represent a valid and efficient method for obtaining a higher quality of recycled tiles from building waste.

## 2. Materials and Methods

### 2.1. Investigated Samples

The studied samples were collected from Cosmari Srl, a stationary recycling plant located in the province of Macerata (Italy), responsible for sorting and managing post-earthquake debris. The samples are constituted by tile and cement mortar coming from collapsed building during the Amatrice earthquake in 2016 and 2017. In more detail, the different fragments consist of pure tile, tile partially or totally covered by cement mortar, and pure cement mortar.

**Figure 1:**
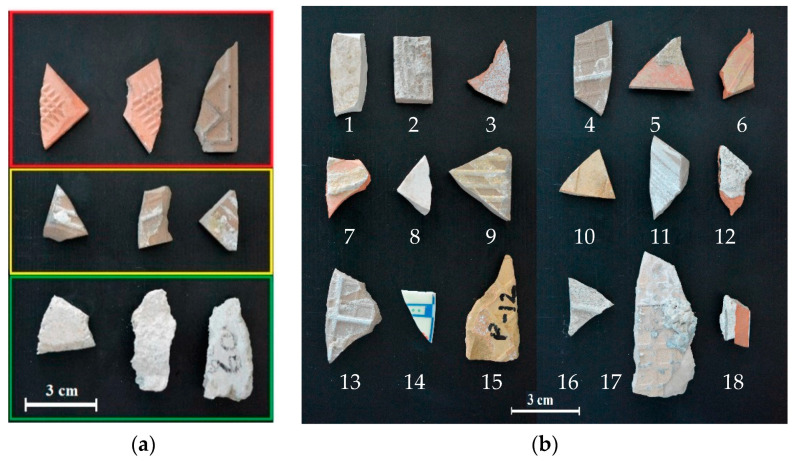
RGB color images of the acquired construction and demolition waste (CDW) samples: (**a**) calibration dataset composed of pure tile (red box), partially covered tile (yellow box), and pure cement mortar fragments (green box) and (**b**) validation dataset composed of pure tiles and partially or totally covered by cement mortar tile.

Samples were divided into calibration and validation datasets (Figure 1), used for the development and application of the HSI classification model.

The calibration dataset was used to build the classification model and consist of three classes of materials: pure tile, partially covered tile, and pure cement mortar fragments (Figure 1a). The validation dataset consists of a mosaic of two acquisitions, containing a mixture of clean tile (1, 6, 8, 10, and 14) and tile partially (3–5, 7, 9, 11–13, 15–18) or totally covered (2) by cement mortar (Figure 1b). Micro-XRF and HSI analyses were carried out at the Raw-Materials Lab of the Department of Chemical Engineering, Materials and Environment (Sapienza—University of Rome, Italy).

### 2.2. Micro-X-ray Fluorescence

To evaluate chemical composition and element distribution, samples were identified and characterized by micro-XRF. Measurements were performed using a M4 Tornado (Bruker) equipped with a Rh tube, operating at 50 kV, 200 μA, with 25 μm spot obtained with poly-capillary optics. All acquisitions were acquired under vacuum conditions at 20 mBar, with a step size of 110 μm and mapping acquisition at 10 ms/pixel. The analyzed elements for the identification of tile from cement mortar are calcium (Ca), silicon (Si), aluminium (Al), and iron (Fe). A mosaic of four-element maps was used for comparison with those obtained by HSI classification.

### 2.3. Hyperspectral Imaging

HSI was carried out in the short-wave infrared range (SWIR: 1000–2500 nm). Images were acquired using the SISUChemaXL^TM^ Chemical Imaging Workstation (Specim, Oulu, Finland), equipped with an ImSpector^TM^ N25E imaging spectrograph (Specim, Oulu, Finland). Acquisitions are controlled by a PC unit equipped with a specialized acquisition/pre-processing software ChemaDAQ^TM^ (Specim, Oulu, Finland) and the sensing device constituting the platform as well as performing spectra acquisition and collection. Hyperspectral images were acquired with a 31 mm lens, with field of view of 10 cm and pixel resolution of 300 µm. Calibration for black and white references was automatically performed. In total, 256 wavelengths were collected.

### 2.4. Data Handling and Analysis

Micro-XRF and HSI derived data were analyzed adopting standard chemometric methods [45,46] by the PLS_Toolbox (Version 8.9 Eigenvector Research, Inc., Manson, WA, USA) [47] running inside Matlab 2020b (The Mathworks, Inc., Natick, MA, USA). In more detail, micro-XRF and HSI data analysis was performed as follows: the raw spectra were pre-processed to reduce unwanted effects and highlight useful information. PCA was then applied for exploratory purposes. Finally, PLS-DA was utilized to create a classification model from HSI data.

#### 2.4.1. Spectra Pre-Processing

Spectra pre-processing is usually performed to reduce undesired physical phenomena, potentially occurring during spectra acquisition, due to sensing device architecture characteristics with respect to environmental constraints, sample attributes (i.e., size, shape, morphology, etc.), or energizing source characteristics (i.e., geometry, stability, etc.) [48]. In particular, the background of each image was removed. Then data were pre-processed to highlight sample spectral differences and to reduce the potential effect of external sources, which can influence collected spectra characteristics.

The pre-processing algorithms applied to micro-XRF data are Standard Normal Variate (SNV) and Autoscale, whereas those selected for HSI data are SNV, first derivative, and Mean Center (MC).

SNV is a weighted normalization and it was utilized to solve scaling, gain effects, scattering effects, source or detector variations, and other general instrumental sensitivity effects [47].

Autoscale uses the mean-center and sets the variance within the data to 1. This method is often used when responses are on different magnitude scales.

Derivative is a useful method for removing baseline signals from samples. The simplest form of a derivative is a point-difference first derivative: each variable in a sample is subtracted from its immediate neighboring variable [48].

MC is one of the most common pre-processing methods: it calculates the mean of each column of the matrix associated with the image and subtracts this from the column. It is useful for removing constant background contributions, which usually are not interesting for data variance interpretation [49].

#### 2.4.2. Principal Components Analysis (PCA)

PCA is a powerful and versatile method capable of providing an overview of complex multivariate data. PCA can be used for revealing relations between variables and samples (e.g., clustering), detecting outliers, finding and quantifying patterns, and generating new hypotheses, among other used [50,51]. It was used to decompose the “processed” spectral data into several principal components (PCs) (linear combinations of the original spectral data) embedding the spectral variations of each collected spectral data set. Using this approach, a reduced set of factors is produced. Such a set can be used for discrimination since it provides an accurate description of the entire dataset. Samples characterized by similar spectra, belonging to the same class of products, are grouped in the same region of the score plot related to the first two or three PCs, whereas samples characterized by different spectral features will be clustered in other parts of this space. A PCA score plot of micro-XRF data from calibration set was used to detect tile and cement mortar materials and, starting from the score images, their percentage was calculated for each fragment. The score image of the validation set was then used to evaluate the correctness of HSI classification. PCA applied to HSI data was used to evaluate the spectral differences between tile and cement mortar materials after applying different pre-processing strategies to select the one that provides the best class separation.

#### 2.4.3. Partial Least Square Discriminant Analysis (PLS-DA)

PLS-DA was applied to HSI data to build a predictive model able to classify tile and cement mortar. This classification technique combines the properties of partial least squares regression with the ability of a classification technique. It is a classification method used to find a model able to predict the known classes in an unknown image [52,53,54]. Prior knowledge of the data was required. Starting from known samples, a distinguishing function is built to predict the new unknown object in the HSI image, made of the same materials of the known classes.

PLS-DA is used to classify samples into predefined groups by forming discriminant functions from input variables (i.e., wavelengths) to yield a new set of transformed values that provides more accurate discrimination than any single variable (i.e., wavelength). A discriminant function is then built using samples with known groups to be employed later to classify samples with an unknown group set. Therefore, once the model is obtained, it can be applied to an entire hypercube and for the classification of a new hypercube. The same pre-processing algorithms used in the PCA step were applied. The result of PLS-DA, applied to the hypercubes, is a “prediction map”, where the classes (i.e., tile and cement mortar) are defined by different colors.

Classification models were then evaluated using the following parameters: Sensitivity and Specificity in calibration (Cal) and cross-validation (CV):

Sensitivity: TP/(TP + FN)(1)

Specificity: TN/(TN + FP)(2)

where TP are true positive, TN true negative and FN false negatives. The best models are obtained when similar values are obtained for Sensitivity and Specificity in Cal and CV, thus demonstrating the robustness of the developed model [55]. Receiver Operating Characteristics (ROC) curves were adopted to evaluate the classification capability of the model. A perfect classification method would yield a point in the upper left corner of the ROC space, representing maximum sensitivity and specificity, while a random classification gives points along the diagonal line from the left bottom to the top right corner [52].

Finally, the prediction results, in terms of pixel percentage (i.e., tile and cement mortar presence in each fragment), obtained by the PLS-DA model were compared with those coming from the micro-XRF map obtained by class set on PC1-PC2 score plot.

## 3. Results and Discussion

### 3.1. Micro-XRF Results

**Figure 2:**
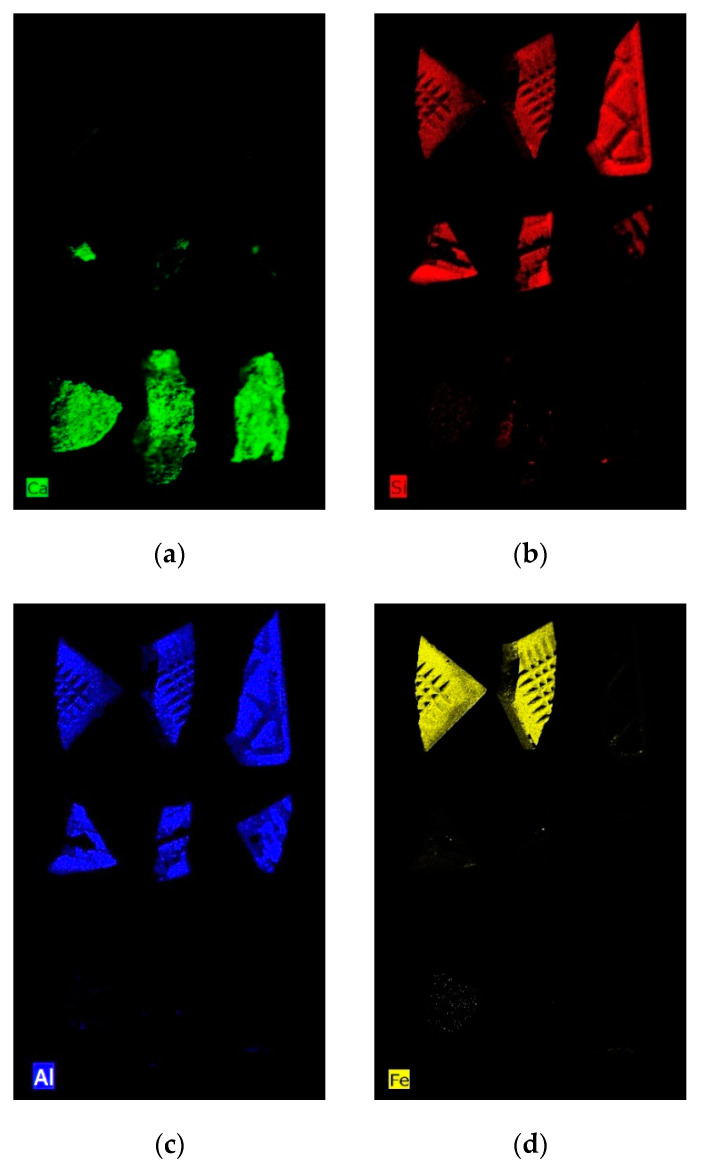
Micro-X-ray fluorescence (micro-XRF) maps of the calibration dataset showing the distribution of representative elements for the two classes of materials: (**a**) Ca, (**b**) Si, (**c**) Al, and (**d**) Fe.

The results of micro-XRF mapping on the calibration set are shown in Figure 2. It was able to highlight the main chemical differences between the two classes of studied materials and to evaluate the distribution on the fragment surface of the selected chemical elements representative of the two classes of materials (i.e., tile and cement mortar). In particular, the maps of Ca and Si are representative of the presence of CaCO_3_ constituting cement mortar and SiO_2_ constituting tile, respectively. Fe and Al allow better identification of tile, as they are present in a very low concentration in the cement mortar.

**Figure 3:**
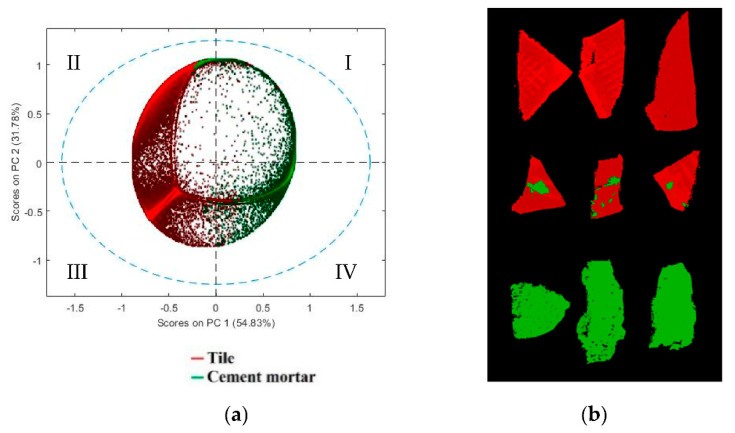
(**a**) Principal component analysis (PCA) score plot utilized to perform micro-XRF data, applying Standard Normal Variate (SNV) and Autoscale pre-processing, and (**b**) image of score representative of the classes set on the PCA score plot.

In order to quantify the percentage of tile and cement mortar on each fragment using micro-XRF maps, all the element maps were concatenated, and a mosaic was created [56]. The resulting dataset was pre-processed by SNV and Autoscale, before the analysis by PCA. PCA model requires three PCs to express a total captured variance of 100%. The PCA score plot (Figure 3a) realizes good discrimination between the two classes. In the PC1-PC2 score plot it is possible to observe that tile pixels can be easily discriminated, being mainly concentrated in the second and third quadrants. Pixels belonging to the cement mortar class occur in different regions of the plot, mainly in the first and fourth quadrants. The score image, with class set, can show the map distribution of cement mortar and tile on fragment surfaces based on micro-XRF analysis (Figure 3b).

### 3.2. Hyperspectral Imaging Results

**Figure 4:**
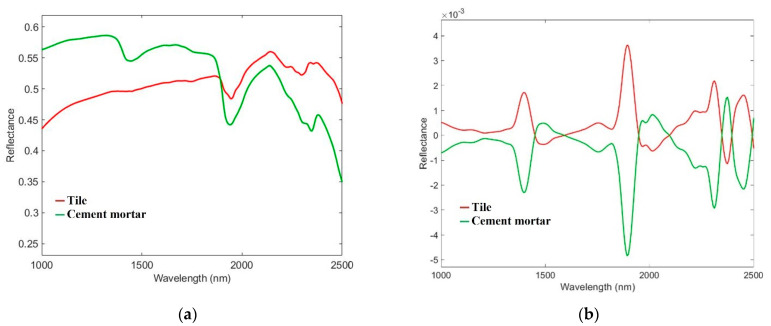
Short-wave infrared range (SWIR) average reflectance spectra of the two classes of materials constituting the calibration dataset (**a**) before and (**b**) after pre-processing.

The average raw reflectance spectra of the analyzed tile and cement mortar materials are reported in Figure 4a. The absorption features of the cement mortar spectra, visible around 1400 nm and 1900 nm, are due to the O-H stretching and H-O-H bending vibrations in the water molecules [57]. Moreover, the absorption at 2350 nm could identify calcite, which is one of the cement mortar ingredients in the form of calcium carbonate [58]. Tile spectra have an accentuated absorption around 2200 nm. This characteristic could be attributed to a higher content of Al as observed by micro-XRF map results. In fact, a combination band related to the Al-O-H bond is detected in the SWIR range between 2180 nm and 2230 nm [59]. The corresponding pre-processed spectra, obtained after the application of SNV, 1st Derivative and MC, are shown in Figure 4b.

**Figure 5:**
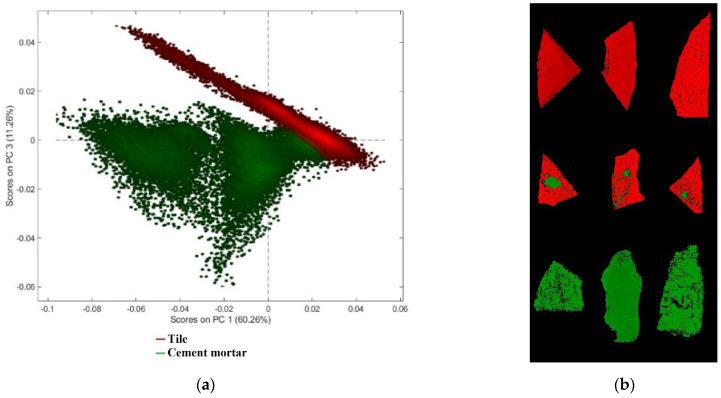
(**a**) Score plot (PC1-PC3) of calibration dataset and (**b**) image of score showing pixels corresponding to the two classes set on the PCA score plot.

PCA model requires four PCs to express a total captured variance of 91.90%. The PC1-PC3 score plot (Figure 5a) shows good discrimination between tile and cement mortar. In particular, the analysis of PC1 and PC3 shows that cement mortar is clustered into two subgroups, due to different morphology of the selected samples, which produces scattering noise. The false color image (Figure 5b) shows fragment pixels corresponding to the two classes set on the PCA score plot.

**Figure 6:**
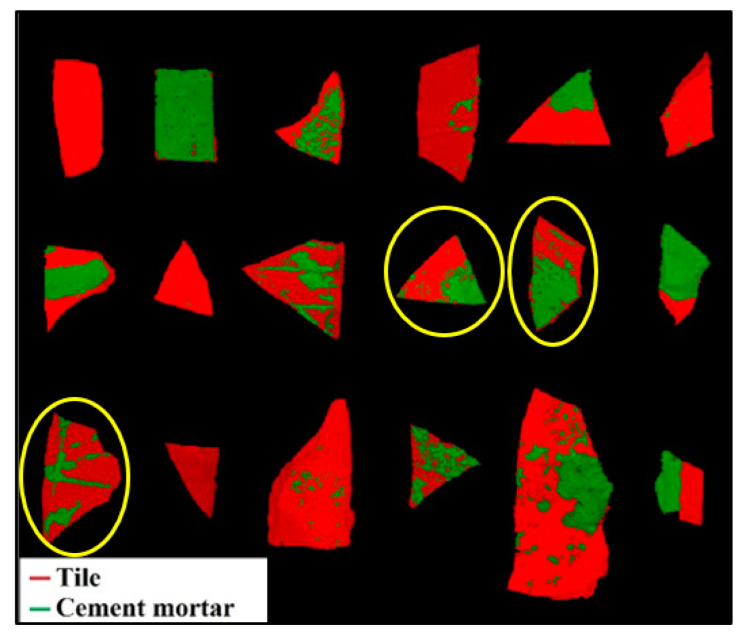
Prediction map resulting from the application of the PLS-DA model, showing the classification of tile and cement mortar materials on the different fragments.

Starting from the dataset calibrated by PCA, a PLS-DA model was created and applied to the validation dataset. The results are shown in Figure 6 in terms of prediction maps. Comparing the prediction map with the corresponding source image (Figure 1b), it is possible to observe a good qualitative recognition of the tile and cement mortar materials, except for samples 10, 11, and 13. These samples are misclassified, due to their non-planar surface which generates noise.

PLS-DA model was evaluated in terms of Sensitivity and Specificity (Table 1). The good quality of the dataset is confirmed by the values of the two parameters, ranging from 0.931 to 0.982 and 0.850 to 0.928 for the two classes of materials respectively, both in calibration and cross-validation.

**Figure 7:**
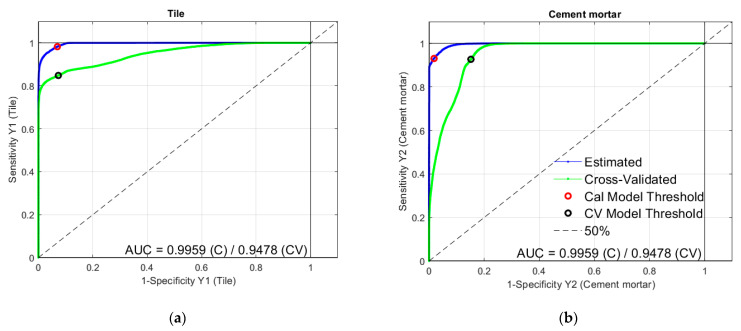
ROC curves for tile (**a**) and cement mortar (**b**) classes.

ROC curves for tile and cement mortar classes are shown in Figure 7. As expected from the results of Table 1, the ROC curves of both classes are of good quality.

### 3.3. Comparison of Micro-XRF Results and HSI Prediction

**Figure 8:**
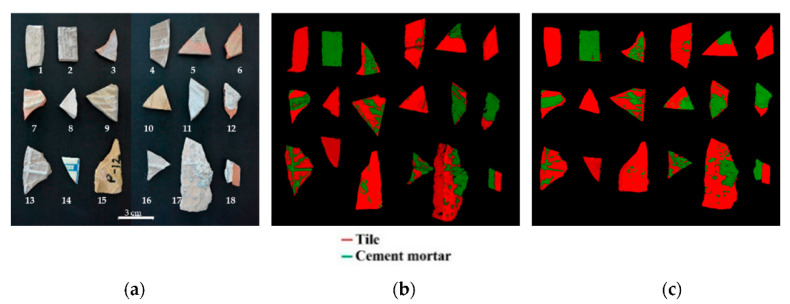
Comparison of results on validation dataset: (**a**) RGB image with samples labels; (**b**) micro-XRF score image of PCA and (**c**) hyperspectral imaging (HSI) prediction map.

The prediction map obtained by the PLS-DA model was compared with the results obtained by micro-XRF, in order to evaluate the correct assignment to the two classes of materials in terms of each constituting pixel (Figure 8). The class population statistics of cement mortar and tile for each fragment obtained by micro-XRF and HSI analysis was quantitatively evaluated, and the results are shown in Table 2. The percentages of pixel distribution among tile and cement mortar for each investigated fragment achieved by HSI prediction are in agreement with those obtained by micro-XRF analysis. This positive result confirms the small classification errors of the PLS-DA model. More in detail, the percentage difference between the two applied techniques ranges from 0 to 8%, except for fragments number 10, 11 and 13, characterized by an error ranging from 14 to 34% for both classes of materials. This is probably due to the morphology of the samples, showing some regions not perfectly in focus. However, the quality of the detection can be considered good, since the PLS-DA model was able to detect most of the pixels corresponding to the cement mortar.

## 4. Conclusions

A methodology was developed and tested for assessing the degree of cement mortar from tile liberation observed in post-earthquake building waste, in order to improve the quality of recycled material. To reach this goal, micro-XRF and HSI techniques were applied to the investigated samples. Micro-XRF was selected to evaluate the developed HSI classification strategy in terms of prediction maps. Results showed that the proposed technology, combined with specific chemometric logics, is particularly suitable for carrying out the identification of cement mortar attached to the tile surface. The correctness of HSI prediction results was confirmed by the comparison with corresponding micro-XRF element maps. The proposed innovative approach, useful for identifying contaminant-free tiles to be reused as RMA, thus reducing costs related to their actual disposal in dumps and environmental pollution, can represent an important analytical/detection tool in a full circular economy and sustainable development perspective. The strategy presented in this study could be implemented at recycling plant scale in order to certify the quality of final products. RMA, achieving a sufficient “degree of liberation”, can be used as aggregates in concrete, thus contributing in terms of secondary raw materials upcycling. The developed procedure can also be useful for the recognition and the further sorting of contaminant elements such as cement mortar, usually difficult to detect in smaller quantities. HSI could allow the development of on-line strategies for sorting and/or quality control and quality assurance of recycled tile products, avoiding the need for laboratory analysis and intermediate storage and, if possible, quality and end-of-waste certification at the site without human intervention.

## Figures and Tables

**Table 1:** Sensitivity and Specificity for the PLS-DA model built to perform tile and cement mortar recognition.

		Class	
		Tile	Cement Mortar
Sensitivity	Calibration	0.982	0.931
	Cross validation	0.931	0.982
Specificity	Calibration	0.850	0.928
	Cross validation	0.928	0.850

**Table 2:** Percentage of pixels belonging to each class (i.e., tile and cement mortar) in every fragment on the prediction image (HSI) and micro-XRF maps.

Fragment Label	Micro-XRF		HSI	
	Tile (pixel %)	Cement Mortar (pixel %)	Tile (pixel %)	Cement Mortar (pixel %)
1	100	0	100	0
2	0	100	4	96
3	45	55	51	49
4	93	7	95	5
5	74	26	70	30
6	99	1	98	2
7	50	50	50	50
8	100	0	100	0
9	61	39	67	33
10	95	5	61	39
11	26	74	44	56
12	20	80	19	81
13	60	40	74	26
14	98	2	99	1
15	90	10	95	5
16	35	65	43	57
17	72	28	73	27
18	52	48	51	49

## Data Availability

Not applicable.
